# 4-Carbamoylpyridin-1-ium 2,2,2-trichloro­acetate

**DOI:** 10.1107/S1600536812035507

**Published:** 2012-08-23

**Authors:** Franc Perdih

**Affiliations:** a Faculty of Chemistry and Chemical Technology, University of Ljubljana, Aškerčeva 5, P. O. Box 537, SI-1000 Ljubljana, Slovenia, and CO EN–FIST, Dunajska 156, SI-1000 Ljubljana, Slovenia

## Abstract

In the asymmetric unit of the title salt, C_6_H_7_N_2_O^+^·C_2_Cl_3_O_2_^−^, there are two crystallographic independent ion pairs. The amide groups of the 4-carbamoylpyridin-1-ium ions are slightly twisted out of the plane of the aromatic ring with C—C—C—N torsion angles of 8.8 (9)° and 4.6 (8)°. In the crystal, the 4-carbamoylpyridin-1-ium ion is N—H⋯O hydrogen bonded to the trichloro­acetate ion *via* the pyridinium unit and amide group. Layers parallel to the *ac* plane are formed due to the N—H⋯O hydrogen bonding of the adjacent amide groups of 4-carbamoylpyridin-1-ium ions. Weak C—H⋯O inter­actions also occur.

## Related literature

For applications of co-crystals, see: Karki *et al.* (2009 ▶); Friščić & Jones (2010 ▶). For related structures, see: Das & Baruah (2011 ▶). 
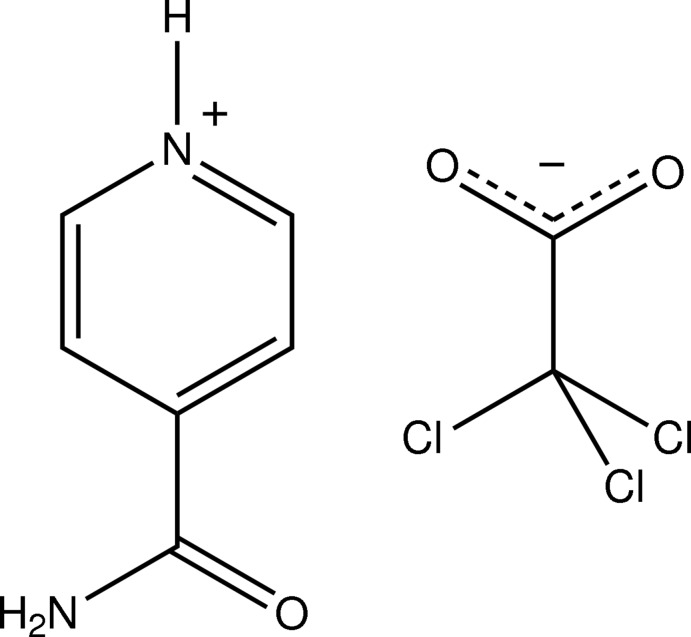


## Experimental

### 

#### Crystal data

C_6_H_7_N_2_O^+^·C_2_Cl_3_O_2_^−^
*M* *_r_* = 285.51
Monoclinic, 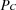

*a* = 9.8768 (3) Å
*b* = 9.4403 (3) Å
*c* = 12.5157 (3) Å
β = 90.240 (2)°
*V* = 1166.95 (6) Å^3^
*Z* = 4
Mo *K*α radiation
μ = 0.78 mm^−1^
*T* = 293 K
0.2 × 0.2 × 0.2 mm

#### Data collection

Agilent SuperNova, Dual, Cu at zero, Atlas diffractometer
Absorption correction: multi-scan (*CrysAlis PRO*; Agilent, 2011 ▶) *T*_min_ = 0.860, *T*_max_ = 0.860
11114 measured reflections
5195 independent reflections
4452 reflections with *I* > 2σ(*I*)
*R*_int_ = 0.026

#### Refinement

*R*[*F* ^2^ > 2σ(*F* ^2^)] = 0.069
*wR*(*F* ^2^) = 0.199
*S* = 1.04
5195 reflections
289 parameters
2 restraints
H-atom parameters constrained
Δρ_max_ = 0.53 e Å^−3^
Δρ_min_ = −0.47 e Å^−3^
Absolute structure: Flack (1983 ▶), 2512 Friedel pairs
Flack parameter: 0.08 (12)

### 

Data collection: *CrysAlis PRO* (Agilent, 2011 ▶); cell refinement: *CrysAlis PRO*; data reduction: *CrysAlis PRO*; program(s) used to solve structure: *SHELXS97* (Sheldrick, 2008 ▶); program(s) used to refine structure: *SHELXL97* (Sheldrick, 2008 ▶); molecular graphics: *ORTEP-3 for Windows* (Farrugia, 1997 ▶) and *DIAMOND* (Brandenburg, 1999 ▶); software used to prepare material for publication: *WinGX* publication routines (Farrugia, 1999 ▶) and *publCIF* (Westrip, 2010 ▶).

## Supplementary Material

Crystal structure: contains datablock(s) global. DOI: 10.1107/S1600536812035507/bq2374sup1.cif

Structure factors: contains datablock(s) I. DOI: 10.1107/S1600536812035507/bq2374Isup2.hkl

Supplementary material file. DOI: 10.1107/S1600536812035507/bq2374Isup3.cml

Additional supplementary materials:  crystallographic information; 3D view; checkCIF report

## Figures and Tables

**Table 1:** Hydrogen-bond geometry (Å, °)

*D*—H⋯*A*	*D*—H	H⋯*A*	*D*⋯*A*	*D*—H⋯*A*
N1—H1*A*⋯O2^i^	0.86	1.78	2.643 (5)	176
N2—H2*A*⋯O1^ii^	0.86	2.03	2.883 (6)	170
N2—H2*B*⋯O6^iii^	0.86	2.06	2.854 (7)	152
N3—H3*A*⋯O3^iv^	0.86	1.8	2.661 (5)	178
N4—H4*A*⋯O4^v^	0.86	2.01	2.858 (6)	169
N4—H4*B*⋯O5^v^	0.86	2.03	2.858 (7)	160
C2—H2⋯O6^iii^	0.93	2.34	3.251 (7)	166
C5—H5⋯O3	0.93	2.6	3.422 (8)	148
C8—H8⋯O5^v^	0.93	2.36	3.270 (6)	166

Symmetry codes: (i) 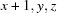; (ii) 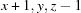; (iii) 
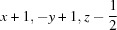
; (iv) 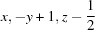; (v) 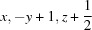.

**Fig. 1.**
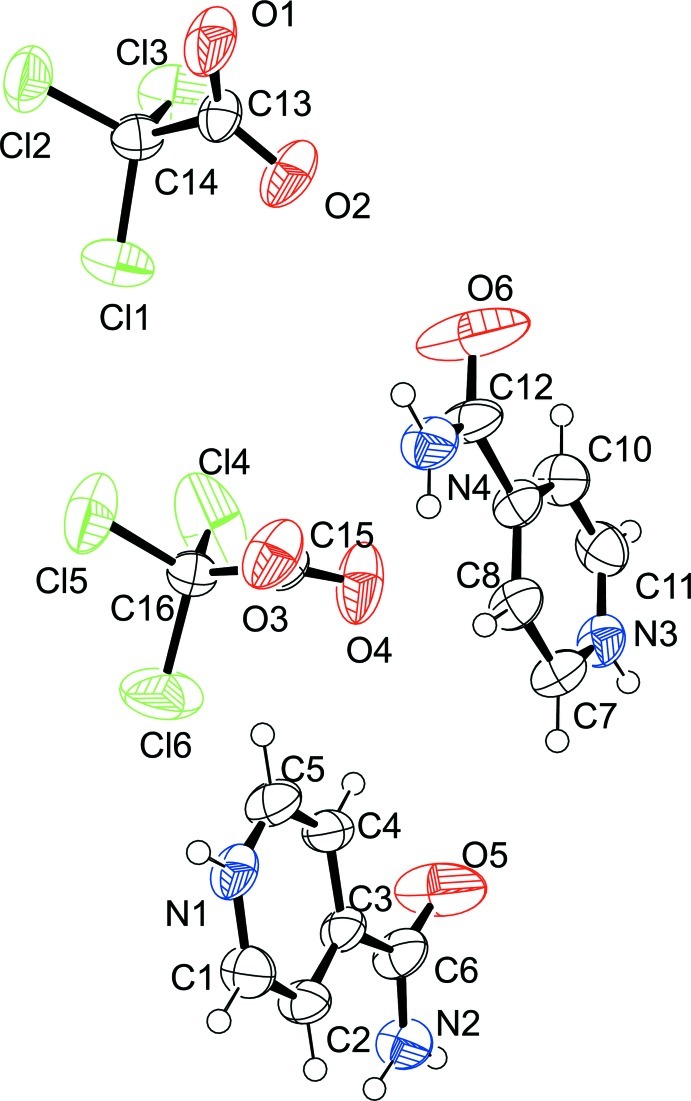
The asymmetric unit of the title compound with displacement ellipsoids drawn at the 50% probability level.

**Fig. 2.**
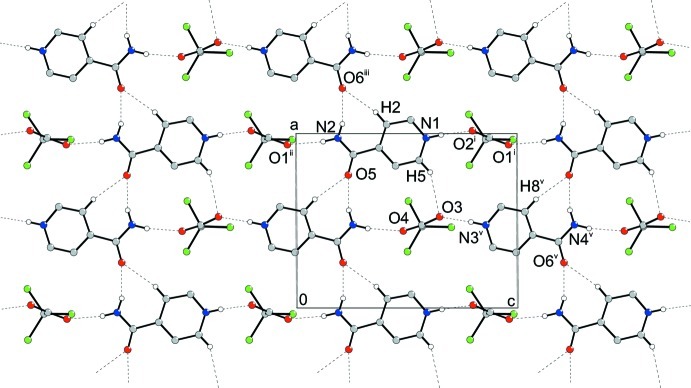
Layer formation. Dashed lines indicate intermolecular N—H···O and C—H···O hydrogen bonding. For the sake of clarity, H atoms not involved in the motif shown have been omitted. Symmetry codes: ix + 1, y, z; iix + 1, y, z – 1; iiix + 1, –y + 1, z – 1/2; vx, –y + 1, z + 1/2.

## supplementary crystallographic information

### Comment

Salts or co-crystals represent two possible ways to produce functional pharmaceutical materials. The salt or co-crystal formation is dependent on a p*K*_a_ difference between the acid and the base (Karki *et al.*, 2009; Friščić & Jones, 2010). Here we present the structure obtained by contacting isonicotinamide and trichloroacetic acid in 1:1 molar ratio.

The asymmetric unit of (I) consists of two crystallographic independent 4-carbamoylpyridin-1-ium cations and two trichloroacetate anions (Fig. 1). The amide groups of 4-carbamoylpyridin-1-ium ions are only slightly twisted out of the plane of the aromatic ring with a C—C—C—N torsion angle of 8.8 (9)° and 4.6 (8)°, respectively. The 4-carbamoylpyridin-1-ium ion is N—H···O hydrogen bonded to the trichloroacetate ion *via* the pyridinium unit and amide group (Fig. 2). Two-dimensional framework is formed due to the N—H···O hydrogen bonding of the adjacent amide groups of 4-carbamoylpyridin-1-ium ions. This layer formation is further stabilized by weak C—H···O interactions. In the structure of (I) typical amide-amide hydrogen-bonded homodimer is not present as is for example in the 4-carbamoylpyridin-1-ium 3-carboxypicolinate (Das & Baruah, 2011).

### Experimental

Crystals of the title compound were obtained by slow evaporation of a 1:1 mol. mixture of isonicotinamide and trichloroacetic acid in methanol at room temperature.

### Refinement

The presence of atoms H1A and H3B bonded to N1 and N3, respectively was confirmed by the observation of peaks in those locations in an electron-density map. All H atoms were then added at calculated positions and refined using a riding model, with C—H = 0.93 Å and N—H = 0.86 Å, with *U*_iso_(H) = 1.2*U*_eq_(C) and 1.5*U*_eq_(N). To improve the refinement results, two reflection with too high value of *δ*(*F*^2^)/e.s.d. and with *F*_o_^2^ < *F*_c_^2^ were deleted from the refinement. Displacement ellipsoid of O5 and O6 are large compared to the other atoms, however the treatment of O5 and O6 as disordered over two positions did not improve the model.

### Crystal data

**Table:**

C_6_H_7_N_2_O^+^·C_2_Cl_3_O_2_^−^	*F*(000) = 576
*M**_r_* = 285.51	*D*_x_ = 1.625 Mg m^−^^3^
Monoclinic, *P**c*	Mo *K*α radiation, λ = 0.71073 Å
Hall symbol: P -2yc	Cell parameters from 4989 reflections
*a* = 9.8768 (3) Å	θ = 3.0–30.3°
*b* = 9.4403 (3) Å	µ = 0.78 mm^−^^1^
*c* = 12.5157 (3) Å	*T* = 293 K
β = 90.240 (2)°	Cube, colourless
*V* = 1166.95 (6) Å^3^	0.2 × 0.2 × 0.2 mm
*Z* = 4	

### Data collection

**Table:**

Agilent SuperNova, Dual, Cu at zero, Atlas diffractometer	5195 independent reflections
Radiation source: SuperNova (Mo) X-ray Source	4452 reflections with *I* > 2σ(*I*)
Mirror monochromator	*R*_int_ = 0.026
Detector resolution: 10.4933 pixels mm^-1^	θ_max_ = 27.5°, θ_min_ = 3.0°
ω scans	*h* = −12→12
Absorption correction: multi-scan (*CrysAlis PRO*; Agilent, 2011)	*k* = −12→12
*T*_min_ = 0.860, *T*_max_ = 0.860	*l* = −16→16
11114 measured reflections	

### Refinement

**Table:**

Refinement on *F*^2^	Secondary atom site location: difference Fourier map
Least-squares matrix: full	Hydrogen site location: inferred from neighbouring sites
*R*[*F*^2^ > 2σ(*F*^2^)] = 0.069	H-atom parameters constrained
*wR*(*F*^2^) = 0.199	*w* = 1/[σ^2^(*F*_o_^2^) + (0.104*P*)^2^ + 1.1455*P*] where *P* = (*F*_o_^2^ + 2*F*_c_^2^)/3
*S* = 1.04	(Δ/σ)_max_ < 0.001
5195 reflections	Δρ_max_ = 0.53 e Å^−^^3^
289 parameters	Δρ_min_ = −0.47 e Å^−^^3^
2 restraints	Absolute structure: Flack (1983), 2512 Friedel pairs
Primary atom site location: structure-invariant direct methods	Flack parameter: 0.08 (12)

### Special details

**Table:**

Geometry. All s.u.'s (except the s.u. in the dihedral angle between two l.s. planes) are estimated using the full covariance matrix. The cell s.u.'s are taken into account individually in the estimation of s.u.'s in distances, angles and torsion angles; correlations between s.u.'s in cell parameters are only used when they are defined by crystal symmetry. An approximate (isotropic) treatment of cell s.u.'s is used for estimating s.u.'s involving l.s. planes.
Refinement. Refinement of *F*^2^ against ALL reflections. The weighted *R*-factor *wR* and goodness of fit *S* are based on *F*^2^, conventional *R*-factors *R* are based on *F*, with *F* set to zero for negative *F*^2^. The threshold expression of *F*^2^ > 2σ(*F*^2^) is used only for calculating *R*-factors(gt) *etc*. and is not relevant to the choice of reflections for refinement. *R*-factors based on *F*^2^ are statistically about twice as large as those based on *F*, and *R*- factors based on ALL data will be even larger.

### Fractional atomic coordinates and isotropic or equivalent isotropic displacement parameters (Å^2^)

**Table:**

	*x*	*y*	*z*	*U*_iso_*/*U*_eq_	
Cl1	0.10761 (17)	0.0588 (2)	0.78518 (16)	0.0826 (5)	
Cl2	−0.0364 (2)	0.03005 (15)	0.98391 (14)	0.0768 (5)	
Cl3	−0.18351 (17)	0.0792 (2)	0.78632 (16)	0.0819 (5)	
Cl4	0.3217 (3)	0.0487 (2)	0.5048 (3)	0.1287 (12)	
Cl5	0.4613 (3)	0.04358 (18)	0.70263 (17)	0.0950 (7)	
Cl6	0.6112 (3)	0.0464 (2)	0.5097 (3)	0.1206 (10)	
N1	0.9738 (5)	0.3469 (4)	0.5899 (3)	0.0465 (9)	
H1A	0.9911	0.3487	0.6573	0.07*	
N2	0.9725 (5)	0.3126 (5)	0.1897 (3)	0.0545 (11)	
H2A	0.9523	0.3112	0.1228	0.082*	
H2B	1.0545	0.2975	0.21	0.082*	
N3	0.4737 (5)	0.6594 (5)	0.3579 (3)	0.0489 (10)	
H3A	0.4908	0.6608	0.2906	0.073*	
N4	0.4753 (5)	0.6746 (5)	0.7577 (3)	0.0519 (10)	
H4A	0.4549	0.6785	0.8243	0.078*	
H4B	0.5587	0.6786	0.7384	0.078*	
O1	−0.0643 (6)	0.3280 (4)	0.9614 (3)	0.0789 (15)	
O2	0.0158 (5)	0.3511 (5)	0.7986 (3)	0.0713 (13)	
O3	0.5208 (6)	0.3362 (5)	0.6487 (3)	0.0713 (12)	
O4	0.4394 (7)	0.3324 (4)	0.4841 (3)	0.0834 (16)	
O5	0.7614 (5)	0.3595 (9)	0.2357 (4)	0.116 (3)	
O6	0.2588 (5)	0.6567 (10)	0.7106 (4)	0.138 (3)	
C1	1.0769 (5)	0.3404 (6)	0.5206 (4)	0.0512 (12)	
H1	1.1657	0.3372	0.5456	0.061*	
C2	1.0507 (5)	0.3384 (6)	0.4112 (4)	0.0451 (10)	
H2	1.1213	0.3352	0.3623	0.054*	
C3	0.9178 (5)	0.3415 (5)	0.3768 (4)	0.0410 (9)	
C4	0.8152 (5)	0.3479 (5)	0.4506 (4)	0.0471 (10)	
H4	0.7253	0.3503	0.4283	0.057*	
C5	0.8467 (6)	0.3506 (6)	0.5575 (4)	0.0555 (13)	
H5	0.7775	0.3551	0.6076	0.067*	
C6	0.8784 (6)	0.3376 (6)	0.2605 (4)	0.0538 (13)	
C7	0.5779 (6)	0.6614 (6)	0.4269 (4)	0.0519 (12)	
H7	0.6667	0.6613	0.4026	0.062*	
C8	0.5502 (5)	0.6637 (5)	0.5346 (4)	0.0470 (11)	
H8	0.6208	0.6664	0.5839	0.056*	
C9	0.4176 (5)	0.6621 (5)	0.5696 (4)	0.0418 (10)	
C10	0.3170 (6)	0.6561 (6)	0.4940 (4)	0.0543 (12)	
H10	0.2271	0.6525	0.5155	0.065*	
C11	0.3464 (6)	0.6552 (6)	0.3887 (4)	0.0556 (13)	
H11	0.2771	0.6518	0.3382	0.067*	
C12	0.3792 (5)	0.6626 (7)	0.6854 (4)	0.0558 (13)	
C13	−0.0250 (5)	0.2831 (5)	0.8761 (3)	0.0436 (10)	
C14	−0.0344 (5)	0.1177 (5)	0.8593 (4)	0.0481 (10)	
C15	0.4722 (5)	0.2781 (5)	0.5682 (3)	0.0419 (9)	
C16	0.4658 (5)	0.1124 (5)	0.5706 (4)	0.0463 (10)	

### Atomic displacement parameters (Å^2^)

**Table:**

	*U*^11^	*U*^22^	*U*^33^	*U*^12^	*U*^13^	*U*^23^
Cl1	0.0647 (9)	0.0950 (12)	0.0882 (12)	0.0167 (8)	0.0213 (8)	−0.0217 (10)
Cl2	0.1036 (12)	0.0537 (7)	0.0730 (10)	0.0026 (8)	0.0071 (9)	0.0188 (7)
Cl3	0.0599 (8)	0.1001 (12)	0.0856 (11)	−0.0164 (8)	−0.0161 (7)	−0.0240 (10)
Cl4	0.1135 (16)	0.0708 (11)	0.201 (3)	−0.0082 (11)	−0.0941 (19)	−0.0250 (14)
Cl5	0.1471 (19)	0.0620 (9)	0.0760 (11)	−0.0091 (10)	0.0003 (12)	0.0210 (8)
Cl6	0.1032 (15)	0.0709 (12)	0.188 (3)	0.0098 (11)	0.0773 (17)	−0.0256 (14)
N1	0.066 (3)	0.049 (2)	0.0250 (16)	−0.0007 (19)	0.0018 (17)	0.0029 (14)
N2	0.059 (2)	0.083 (3)	0.0216 (17)	0.008 (2)	−0.0014 (16)	−0.0018 (19)
N3	0.065 (3)	0.053 (2)	0.0280 (18)	0.004 (2)	−0.0005 (17)	0.0013 (16)
N4	0.057 (2)	0.069 (3)	0.0296 (19)	0.003 (2)	−0.0002 (17)	−0.0002 (18)
O1	0.146 (5)	0.059 (2)	0.0319 (18)	0.012 (3)	0.008 (2)	0.0011 (16)
O2	0.116 (4)	0.071 (3)	0.0270 (16)	−0.017 (2)	0.002 (2)	0.0035 (16)
O3	0.117 (4)	0.065 (2)	0.0324 (18)	−0.017 (2)	0.004 (2)	−0.0052 (16)
O4	0.158 (5)	0.052 (2)	0.040 (2)	0.007 (3)	−0.014 (3)	0.0037 (17)
O5	0.049 (2)	0.251 (8)	0.047 (2)	0.012 (4)	−0.0102 (19)	0.015 (4)
O6	0.051 (3)	0.312 (11)	0.051 (3)	−0.018 (4)	0.011 (2)	0.008 (4)
C1	0.047 (2)	0.067 (3)	0.039 (2)	0.005 (2)	−0.005 (2)	0.001 (2)
C2	0.040 (2)	0.065 (3)	0.030 (2)	0.003 (2)	0.0028 (17)	0.0013 (19)
C3	0.043 (2)	0.052 (3)	0.0288 (18)	−0.0013 (19)	0.0020 (16)	0.0073 (16)
C4	0.040 (2)	0.057 (3)	0.044 (2)	0.001 (2)	0.0088 (19)	0.003 (2)
C5	0.061 (3)	0.065 (3)	0.041 (3)	0.000 (2)	0.018 (2)	0.004 (2)
C6	0.051 (3)	0.082 (4)	0.029 (2)	−0.007 (3)	−0.0046 (19)	0.010 (2)
C7	0.053 (3)	0.063 (3)	0.040 (2)	−0.008 (2)	0.010 (2)	0.004 (2)
C8	0.049 (3)	0.063 (3)	0.029 (2)	−0.008 (2)	−0.0054 (19)	0.0021 (19)
C9	0.047 (2)	0.051 (3)	0.0277 (19)	−0.0028 (19)	−0.0021 (17)	0.0005 (17)
C10	0.049 (3)	0.071 (3)	0.043 (2)	0.002 (2)	−0.005 (2)	−0.001 (2)
C11	0.056 (3)	0.065 (3)	0.046 (3)	0.012 (3)	−0.010 (2)	−0.001 (2)
C12	0.044 (2)	0.093 (4)	0.030 (2)	−0.004 (3)	0.0067 (19)	0.003 (2)
C13	0.056 (2)	0.046 (2)	0.028 (2)	−0.001 (2)	−0.0072 (18)	0.0027 (17)
C14	0.047 (2)	0.051 (3)	0.046 (2)	−0.002 (2)	0.0039 (19)	−0.006 (2)
C15	0.058 (2)	0.044 (2)	0.0239 (18)	−0.004 (2)	0.0062 (17)	−0.0036 (17)
C16	0.044 (2)	0.048 (2)	0.047 (2)	0.001 (2)	0.0028 (19)	−0.005 (2)

### Geometric parameters (Å, º)

**Table:**

Cl1—C14	1.774 (5)	O5—C6	1.213 (7)
Cl2—C14	1.766 (5)	O6—C12	1.233 (7)
Cl3—C14	1.768 (5)	C1—C2	1.393 (6)
Cl4—C16	1.748 (5)	C1—H1	0.93
Cl5—C16	1.777 (6)	C2—C3	1.380 (7)
Cl6—C16	1.743 (5)	C2—H2	0.93
N1—C5	1.318 (7)	C3—C4	1.375 (6)
N1—C1	1.342 (7)	C3—C6	1.505 (6)
N1—H1A	0.86	C4—C5	1.372 (8)
N2—C6	1.308 (7)	C4—H4	0.93
N2—H2A	0.86	C5—H5	0.93
N2—H2B	0.86	C7—C8	1.377 (7)
N3—C11	1.317 (7)	C7—H7	0.93
N3—C7	1.340 (7)	C8—C9	1.382 (7)
N3—H3A	0.86	C8—H8	0.93
N4—C12	1.314 (7)	C9—C10	1.371 (7)
N4—H4A	0.86	C9—C12	1.499 (6)
N4—H4B	0.86	C10—C11	1.350 (8)
O1—C13	1.213 (6)	C10—H10	0.93
O2—C13	1.233 (6)	C11—H11	0.93
O3—C15	1.242 (6)	C13—C14	1.578 (7)
O4—C15	1.215 (6)	C15—C16	1.566 (7)
			
C5—N1—C1	121.8 (4)	C7—C8—C9	120.2 (4)
C5—N1—H1A	119.1	C7—C8—H8	119.9
C1—N1—H1A	119.1	C9—C8—H8	119.9
C6—N2—H2A	120	C10—C9—C8	117.8 (4)
C6—N2—H2B	120	C10—C9—C12	118.8 (4)
H2A—N2—H2B	120	C8—C9—C12	123.4 (4)
C11—N3—C7	122.9 (4)	C11—C10—C9	121.1 (5)
C11—N3—H3A	118.6	C11—C10—H10	119.5
C7—N3—H3A	118.6	C9—C10—H10	119.5
C12—N4—H4A	120	N3—C11—C10	119.6 (5)
C12—N4—H4B	120	N3—C11—H11	120.2
H4A—N4—H4B	120	C10—C11—H11	120.2
N1—C1—C2	119.8 (5)	O6—C12—N4	121.5 (5)
N1—C1—H1	120.1	O6—C12—C9	119.7 (5)
C2—C1—H1	120.1	N4—C12—C9	118.8 (4)
C3—C2—C1	118.6 (4)	O1—C13—O2	128.1 (5)
C3—C2—H2	120.7	O1—C13—C14	116.4 (4)
C1—C2—H2	120.7	O2—C13—C14	115.4 (4)
C4—C3—C2	119.6 (4)	C13—C14—Cl2	110.3 (3)
C4—C3—C6	117.6 (4)	C13—C14—Cl3	108.7 (3)
C2—C3—C6	122.8 (4)	Cl2—C14—Cl3	110.3 (3)
C5—C4—C3	119.5 (5)	C13—C14—Cl1	109.5 (3)
C5—C4—H4	120.3	Cl2—C14—Cl1	109.1 (3)
C3—C4—H4	120.3	Cl3—C14—Cl1	108.9 (3)
N1—C5—C4	120.7 (5)	O4—C15—O3	128.1 (5)
N1—C5—H5	119.6	O4—C15—C16	115.3 (4)
C4—C5—H5	119.6	O3—C15—C16	116.2 (4)
O5—C6—N2	122.4 (5)	C15—C16—Cl6	108.4 (3)
O5—C6—C3	119.0 (5)	C15—C16—Cl4	111.6 (4)
N2—C6—C3	118.6 (5)	Cl6—C16—Cl4	110.0 (3)
N3—C7—C8	118.4 (5)	C15—C16—Cl5	112.6 (3)
N3—C7—H7	120.8	Cl6—C16—Cl5	107.4 (3)
C8—C7—H7	120.8	Cl4—C16—Cl5	106.8 (3)
			
C5—N1—C1—C2	0.5 (8)	C7—N3—C11—C10	1.5 (8)
N1—C1—C2—C3	−0.8 (8)	C9—C10—C11—N3	0.4 (9)
C1—C2—C3—C4	0.7 (8)	C10—C9—C12—O6	0.5 (10)
C1—C2—C3—C6	−179.1 (5)	C8—C9—C12—O6	−177.8 (7)
C2—C3—C4—C5	−0.3 (8)	C10—C9—C12—N4	−177.1 (6)
C6—C3—C4—C5	179.6 (5)	C8—C9—C12—N4	4.6 (8)
C1—N1—C5—C4	0.0 (8)	O1—C13—C14—Cl2	−23.3 (6)
C3—C4—C5—N1	−0.1 (8)	O2—C13—C14—Cl2	159.9 (4)
C4—C3—C6—O5	9.9 (9)	O1—C13—C14—Cl3	97.8 (5)
C2—C3—C6—O5	−170.3 (7)	O2—C13—C14—Cl3	−79.1 (5)
C4—C3—C6—N2	−171.0 (5)	O1—C13—C14—Cl1	−143.3 (5)
C2—C3—C6—N2	8.8 (9)	O2—C13—C14—Cl1	39.8 (6)
C11—N3—C7—C8	−2.1 (8)	O4—C15—C16—Cl6	80.2 (6)
N3—C7—C8—C9	0.8 (8)	O3—C15—C16—Cl6	−93.8 (5)
C7—C8—C9—C10	0.9 (8)	O4—C15—C16—Cl4	−41.1 (6)
C7—C8—C9—C12	179.2 (5)	O3—C15—C16—Cl4	145.0 (5)
C8—C9—C10—C11	−1.6 (9)	O4—C15—C16—Cl5	−161.2 (5)
C12—C9—C10—C11	−180.0 (5)	O3—C15—C16—Cl5	24.9 (6)

### Hydrogen-bond geometry (Å, º)

**Table:**

*D*—H···*A*	*D*—H	H···*A*	*D*···*A*	*D*—H···*A*
N1—H1*A*···O2^i^	0.86	1.78	2.643 (5)	176
N2—H2*A*···O1^ii^	0.86	2.03	2.883 (6)	170
N2—H2*B*···O6^iii^	0.86	2.06	2.854 (7)	152
N3—H3*A*···O3^iv^	0.86	1.8	2.661 (5)	178
N4—H4*A*···O4^v^	0.86	2.01	2.858 (6)	169
N4—H4*B*···O5^v^	0.86	2.03	2.858 (7)	160
C2—H2···O6^iii^	0.93	2.34	3.251 (7)	166
C5—H5···O3	0.93	2.6	3.422 (8)	148
C8—H8···O5^v^	0.93	2.36	3.270 (6)	166

Symmetry codes: (i) *x*+1, *y*, *z*; (ii) *x*+1, *y*, *z*−1; (iii) *x*+1, −*y*+1, *z*−1/2; (iv) *x*, −*y*+1, *z*−1/2; (v) *x*, −*y*+1, *z*+1/2.
